# Efficacy of the commercial plant products acting against influenza-a review

**DOI:** 10.1186/s43094-021-00385-2

**Published:** 2021-12-14

**Authors:** A. Brindha Devi, R. Sarala

**Affiliations:** Department of Botany, Periyar EVR College (Autonomous), (Affiliated to Bharathidasan University, Trichy-24), Trichy, 620 023, Tamil Nadu India

**Keywords:** Influenza, Phytochemicals, Extracts, Commercial plant products, Pharmaceuticals, Anti-influenza

## Abstract

**Background:**

Influenza infection always poses a threat to human and animal health. Vaccines and antiviral drugs are recommended to deal with the situation. The drawback of these remedial agents made the scientist change their focus on an alternative therapy. The anti-influenza effects of plants have been extensively studied, and many pharmaceutical companies have prepared their products on this basis.

**Main body:**

The present review documents the successfully launched anti-influenza commercial products. In specific, it exposes the scientifically validated and evidence-based supporting inhibitory action of influenza and its strains.

**Conclusion:**

This review highlighted the efficacy of the commercial products which effectively combat influenza. It provides a complementary strategy to deal with the worst-case scenario of flu. Meanwhile, to face the emerging strains, brand new products are in great necessity besides prevailing and available drugs.

## Background

Plants enriched with bioactive compounds are always incorporated with therapeutic values. They become an integrative part of traditional medicine by using as a whole or part of it [1]. Until now, 35,000–70,000 plant species have been analyzed for their medicinal use [2]. It is notified that 50% of clinically used drugs are derived from plant sources [3]. Indeed, traditional medicines fulfill the need for primary health care and manage dreadful chronic diseases [4]. Besides that, 80% of the world population in developing countries still relies on traditional medicine practice [5].

Influenza is an acute respiratory illness caused by influenza viruses [6]. Globally, influenza virus infections present a potential threat to human and animal health due to frequent reassortant and novel mutant strains [7]. Human influenza viruses are classified into A, B, and C related to epidemics [8], of which A and B are notified for recurrent annual epidemics, whereas A may create occasional destructive pandemics [9]. The World Health Organization estimates 3–5 million severe illness cases and 290,000–to 650,000 respiratory-related death worldwide due to annual epidemic [10]. In a pandemic, excess morbidity and mortality may occur, consequently resulting in the decline of basic health care provisions [11].

Vaccination is the primary method opted for to prevent influenza infections. Every year new vaccine formulations are recommended by World Health Organization [12]. An official declaration of vaccine production by WHO [13] and marketing vaccine for new emerging strains [14] is the bottleneck of vaccine preparation.

Antiviral drugs are the other option available for the treatment of influenza, which falls under three categories as M2 inhibitors, neuraminidase inhibitors, and nucleoprotein inhibitors [15] targeting the viral components, thwarted by newly emerging strains. So, these viral-specific drugs are not able to become viral resisting drugs for new strains.

To overcome these obstacles, alternative approaches like traditional and complementary medicine are highly commenced. Recent studies give supporting evidence that natural products like extracts and other compounds derived from traditional medicinal plants have a broad spectrum of anti-influenza activities [16, 17]. Many pharmacological companies pay special attention to commercialize natural products because of their extensive functional abilities [18]. Based on these premises, this review emphasizes the commercialized plant products extensively showing effective inhibitory activity against influenza viruses.

## Main text

Influenza virus genetic recombination capabilities evolve rapidly and produce new strains [19]. The continual emergence of influenza viruses remains the main threat to human health results in a considerable record of morbidity and mortality. The seasonal outbreaks result in 90% of death among the elderly [20]. Moreover, influenza attributes to other complications like pneumonia and its related secondary complaints even after the infection [21].

Among the influenza viruses, types A and B cause severe tragic effects in humans. Among them, A not only spreads in humans but also spreads in other species, such as birds, poultry, waterfowl, and mammals [22], and its evolutionary genes are two to three times faster than B [23].

Influenza A viruses are classified into several subtypes based on the structural proteins hemagglutinin (HA/H) and neuraminidase (NA/N), such as H1–H18 and N1–N11 [24]. The subtypes H1–H16 and N1–N9 were identified in wild birds and found as the reservoir and resource of spreading [25]. However, recently the subtypes H17N10 and H18N11 were concordantly recognized in fruit bats [26]. In general, H1, H2, H3, N1, and N2 subtypes extensively dispersed among humans [27]. Transmission of the virus from the other host and genetic reassortment create a pandemic threat in humans. Avian influenza viruses subtypes H5, H7, and H9 produce the daunting effect of the pandemic in humans [28]. Humans are the host for influenza B viruses notified for one-third of mortality with low risk of a pandemic and are not classified into subtypes but with two lineages notified as B/Yamagata and B/Victoria [29].

In recent years, medicinal plants have become an assuring preference for alternate therapy or additional additives to the synthetic anti-influenza drugs [30]. Indeed, 25% of general medicine contains compounds that are isolated from plants [31]. The anti-influenza activities of the phytochemical constituents of the plants have also been documented [32]. Modern pharmaceutical companies keep an eye on these natural phytochemicals, plant extract active against influenza strains [33], and practicing the effort to produce commercial products. Worldwide these commercial products show appreciable effects against the dampen condition arise due to influenza. This review provides comprehensive encapsulated information regarding the inhibitory action of commercial products against influenza viral strains.

### Commercial plant products

Plant products are the most important source of lead in drug discovery and development [34]. In general, they are safer with fewer side effects than synthetic drugs. Hence, pharmaceutical companies utilize plant extracts or phytochemicals to combat vulnerable diseases [35] like influenza. Besides this, the plant-based product offers a new commercial avenue for the pharmaceutical company. Even though the commercial application requires effort and time, they play an inevitable role in reducing the ailment of the disease [36]. The commercial products like COLD-FX®, CYSTUS052®, Echinaforce®, EPs® 7630, Ladania067®, Mentofin®, Oximacro®, Sambucol®, Rubini®, and CJ-E® act synergistically as an immune stimulant and inhibitory action towards influenza. Table 1 List the anti-influenza efficacy of the above-mentioned commercial products.

**Table 1:** List of commercial products active against influenza

S.no.	Commercial product name	Plant name	Plant part used for extract	Rich Bioactive compound	Company	Influenza type	Influenza strains	Reference
1	COLD-FX® (CVT-E002)	*Panax quinquefolium* L	Root	Poly-furanosyl-pyranosyl-saccharide	Afexa Life Sciences Inc., Edmonton, Alberta, Canada	Influenza A H1N1,H3N2 and B	A/Johannesburg/82/96 (H1N1), A/Nanchang/93/95 (H3N2), B/Harbin/07/94	[42]
2	CYSTUS052®	*Cistus incanus* L	Aerial parts	Polyphenol: Flavan-3-ols and proanthocyanidins	Dr. Pandalis NatUrprodukte GmbH & Co. KG, Glandorf, Germany	Influenza A H7N7,H1N1,H5N1	A/FPV/Bratislava/79, (H7N7), A/PuertoRico/8/34(H1N1), A/Thailand/1(KAN1)/2004 (H5N1)	[45, 46]
3	Echinaforce®	*Echinacea purpurea*	Aerial parts and roots	Not mentioned	A. Vogel Bioforce AG, Roggwil, Switzerland	Influenza A H3N2	Not mentioned	[47]
						Influenza A H3N2,H5N1,H7N7,H1N1	A/Victoria/3/75 (H3N2), A/Thailand/KAN-1/2004 (H5N1), A/FPV/Bratislava/79 (H7N7), and A/PuertoRico/8/34, A/Hamburg/1/09 (H1N1)	[48]
						Influenza A H1/H3,and B	Not mentioned	[7]
						Influenza A H9N2	A/chicken/Iran/772/1998 (H9N2)	[49, 50]
						Influenza A H7N9	A/Anhui/1/2013(H7N9)	[52]
						Influenza A H3N2	A/Victoria/H3N2 (H3N2)	[53]
4	EPs® 7630 (Umckaloabo®)	*Pelargonium sidoides* DC	Roots	Polyphenol: Prodelphinidin, gallocatechin and epigallocatechin	Dr. Willmar Schwabe GmbH & Co. KG, Karlsruhe	Influenza A, H1N1, H3N2, H5N1	A/New Caledonia/20/99 (H1N1), A/California/7/2004 (H3N2), A/Thailand/1(Kan1)/04 (H5N1), A/PuertoRico/8/34NS116-GFP (H1N1), A/Luxembourg/46/2009 (H1N1), A/PuertoRico/8/34 (H1N1),A/Luxembourg/663/2008(H1N1), A/Luxembourg/572/2008 (H1N1) A/Luxembourg/01/2005 (H3N2)	[55, 56]
5	Ladania067®	*Ribes nigrum* L	Leaves	Not mentioned	Dr. Pandalis NatUrprodukte GmbH & Co. KG, Glandorf, Germany	Influenza A, H1N1,H1N1 rec, H1N1 pan, H7N7	A/Regensburg/D6/09 (H1N1) A/Puerto Rico/8/34 (H1N1rec), and A/Nordrhein-Wesfalen/173/2009 (H1N1pan) harboring the oseltamivir resistance mutation NA (H275Y), A/FPV/Bratislava/79 (H7N7)	[57, 58]
6	Mentofin®	Eucalyptus-peppermint essential oil	Leaves	Not mentioned	EWABO, Wietmarschen, Germany	Influenza A H9N2	Not mentioned	[63–66]
7	Oximacro®	*Vaccinium macrocarpon* *Aiton*	Fruit	PAC-A dimers and trimers	Biosfered S.r.l., Turin, Italy	Influenza A H1N1 and B	A/Puerto Rico/8/34 (H1N1), B/Lee/40 (B)	[67]
8	Sambucol®	*Sambucus nigra L*	Fruit, standardized black elderberry extract	Flavonoids (sam4,8)	Razei Bar Company in Jerusalem, Israel	Influenza A H1N1(human and animal strains) H3N2 and B	A/Texas 36/91, A/Singapore 6/86 (H1N1 human strains), A/Shangdong 9/93, A/Beijing 32/92(H3N2), B/Panama 45/90, B/Yamagata16/88, B/Ann Arbor 1/86(B) (A/Sw/Ger 2/81, A/Tur/Ger 3/91, and A/Sw/Ger 8533/91(H1Nanimal strains)	[68, 69]
						Avian Influenza A H5N1	H5N1 (NIBRG-14)	[70]
					PharmaCare Europe Ltd., West Sussex, UK	Influenza A H9N2	A/chicken/Iran/772/1998 (H9N2)	[49, 50]
	Rubini®	*Sambucus nigra L*	Fruit, standardized black elderberry extract	Not mentioned	BerryPharma AG Leichlingen, Germany	Human Influenza A H5N1and B	A/Thailand/KAN-1/2004 (H5N1) and B/Massachusetts/71	[73]
	CJ-E® (Concentrated elderberry juice)	*Sambucus nigra L*	Fruit, standardized black elderberry extract	High molecular weight compounds with acidic polysaccharides	Bayernwald, Hengersberg, Germany	Influenza A H1N1	A/NWS/33 (H1N1)	[74]

## COLD-FX® (CVT-E002)

### Brief view

COLD-FX® (CVT-E002), a patented and proprietary extract rich in poly-furanosyl-pyranosyl-saccharide isolated from the roots of *Panax quinquefolium* L. [37]. It contains 80% polysaccharides and oligosaccharides, and 10% of proteins create immunomodulating effects by stimulating the immune system by increasing T-helper and NK cell number [38] and enhance IL-2 and IFN-γ production [39]. Afexa Life Sciences Inc., (formerly CV Technologies, Inc.), Edmonton, Alberta, Canada, is the proprietor for COLD-FX® (CVT-E002) preparation [37, 40].

### Mode of action

McElhaney et al. conducted two randomized, double-blind, placebo-controlled 8- and 12-week trials, during the two influenza seasons in particular 2000 and 2000–2001 enrolled with 90% vaccinated institutionalized elderly participants of 89 and 109, respectively. The participants were orally administered 200 mg of COLD-FX® (CVT-E002) or a placebo twice daily. The result reveals that laboratory-confirmed influenza illness (LCII) and influenza illness were greater in the placebo than COLD-FX® (CVT-E002) group. The investigators concluded that CVT-E002 is safe, effective, and endurable against acute respiratory infections (ARI) for elderly adults in specific [41].

McElhaney and other investigators tested the efficacy of COLD-FX® (CVT-E002) to the National Hockey League Players of the Edmonton Oilers team. A total of 37 volunteers were selected and divided into two groups, comprised of 18 and 19 members. For the first group of 18 members, COLD-FX of 400 mg was supplemented for 30 days, and the second group of 19 members was devoid of supplements. Peripheral blood leukocytes (PBLs) isolated from the blood sample of two groups were cultured with influenza strains A/Johannesburg/82/96, A/Nanchang/93/95, B/Harbin/07/94 and observed the cell-mediated immune response like enhanced release of cytokines TNF-α and IL-2 with moderate granzyme-B production of about 55% in the first group. The second group does not show a significant increase in cytokine level, but granzyme-B production notified as 80% [42].

The same author, along with other investigators, examined the effectiveness of COLD-FX® (CVT-E002) in the prevention of upper respiratory infections (URIs) due to influenza and other viruses to the influenza-vaccinated community-dwelling adults. A multicenter, randomized, double-blind, placebo-controlled trial was carried out by choosing 783 volunteers and divided them into three groups, of which the first received placebo, the second and third groups administered with CVT-E002, with 400 and 800 mg for 6 months. Primary and secondary analyses were performed based on laboratory-confirmed clinical URIs (LCCUs), and Jackson confirmed URIs. The LCCUs analysis showed 5.5%, 5.2%, and 4.6% in the placebo, CVT-E002-400, and CVT-E002-800 group, and the Jackson confirmed URIs analysis brings out 28.9%, 20.0%, and 19.4% for the placebo, CVT-E002-400, and CVT-E002-800 groups severity and illness reduction, due to URIs. In both analyses, CVT-E002 of 800 mg showed a significant ample role in the prevention of respiratory infections in vaccinated seniors [43].

Predy et al. described the immune-modulating effects of daily supplementation of COLD-fx during the onset of the influenza season in 323 healthy adults of age between 18 and 65 years with two times cold affected in the previous year. A randomized double-blind placebo-controlled study was conducted by administered two capsules of COLD-fx for four months. From the 323 volunteers, 21 COLD-fx and placebo administered blood samples were analyzed. In that, T-helper and natural killer (NK) cells counting are in elevated level with a reduced level of IgA in plasma. Hence, the authors concluded that the synergetic action of cells strengthens the immune system, thereby reducing the duration and severity of URIs in COLD-fx administered groups [38].

## CYSTUS052®

### Brief view

CYSTUS052® is enriched with a unique variety of complex, highly polymeric polyphenols, such as flavan-3-ols and proanthocyanidins obtained from the aerial parts of *Cistus incanus* L. This product carried 26% polysaccharide and 25 monomeric components like gallic acid, gallocatechin, catechin, and epicatechin. Dr. Pandalis NatUrprodukte GmbH & Co. KG, Glandorf, Germany is the proprietor, developed CYSTUS052® [44–46].

### Mode of action

The commercialized plant extract CYSTUS052®of *Cistus incanus* L. has potent anti-influenza activity against H7N7 Avian influenza virus (A/FPV/Bratislava/79), human influenza virus A/Puerto Rico/8/34(H1N1), A/Thailand/1(KAN-1)/2004(H5N1), and even in human rhinovirus serotype 14 (HRV14). Ehrhardt et al. scrutinized the effect and observed incapable of virus resistance and reduction of progeny on treating against the extract. In the viral growth on A549 or MDCK cell cultures viability, metabolism is not affected at the effective dose of 50 μg/ml extract. CYSTUS052® polyphenol components bind to the virus–cell surface protein HA, thereby inhibiting the binding with cellular receptors thus entry is prohibited [45].

Droebner et al. performed both in vitro and in vivo studies by using the aerosol formulation of CYSTUS052® and showed its anti-influenza efficacy against H7N7 Avian influenza virus (A/FPV/Bratislava/79). The in vitro studies revealed that pre-treated MDCK cells show a 90% reduction of plaque numbers. For in vivo investigation, virus-infected Balb/c mice are treated with CYSTUS052 extract through aerosol and observed the mice without clinical disease symptoms like weight reduction, elevation in body temperature, and decline in gross motor activity. Hematoxylin and eosin staining of the lungs of mice showed no alterations in the epithelial cells, and the viral receptors sialic acids, α-2, 6 and α-2, 3 expressions also did not show significant changes [46].

## Echinaforce®

### Brief view

Echinaforce® (EF) is a standardized ethanol extract of *Echinacea purpurea* herb and root (95:5) produced by A. Vogel Bioforce AG of Rogueville, Switzerland, which contains potent antiviral compounds [47].

### Mode of action

Respiratory viruses that cause upper respiratory tract infections induce a variety of pro-inflammatory cytokines. Sharma et al. updated the effect of EF against respiratory viruses including influenza of H3N2 strain, rhinoviruses 1A and 14, respiratory syncytial virus, adenovirus types 3 and 11, and herpes simplex virus and feline calicivirus and observed the direct inhibitory activity inhibiting growth and cytokines. The extract at the concentration of 40 μg/ml revealed nearly entire inhibitory activity on the emerged cytokines like IL-6 and IL-8 evoked by the viruses [47].

Pleschka et al. revealed Echinaforce® (EF) at the concentration of 1.6 mg/ml effectively inhibit H3N2 (human strain A/Victoria/3/75), H5N1 (A/Thailand/KAN-1/2004), H7N7 (A/FPV/Bratislava/79), and H1N1 (A/Puerto Rico/8/34, A/Hamburg/1/09) virus infectivity of about 99%. During the direct contact between the extract and the virus, rather than during the pre-infection or post-infection period, a significant inhibitory activity that inhibits the early stages of replication was exposed. Moreover, EF blocks virus HA activity, thereby preventing the entry of the virus into the cells. EF did not develop the resistant strains compared with Tamiflu® even Tamiflu® resistant viruses are susceptible to EF [48].

Jawad et al. conducted a double-blind, randomized, and placebo-controlled trial by administering Echinaforce® to 355 and placebo for 362 participants over four months. During the tenure, 54 and 74 viral infections with 24 and 47 membranous infections including Influenza A (H1/H3) and B, respiratory syncytial virus, coronavirus (229E/OC43/NL63/HKU1), parainfluenza virus (1–4), human metapneumovirus, entero-rhinovirus, adenovirus, and human bocavirus have been detected along with 14 and 34 recurring viral infections recorded for EF and placebo group. On compared with the placebo-treated, infectious agents and infection duration was relatively low for the EF group (*P* < 0.05). Hence, the author recommends long-term usage of EF as the best prophylactic agent for preventive and recurrent viral infections [7].

Karimi et al. analyzed the effect of Echinaforce® (EF), Sambucol® (SAM), and amantadine in the allantoic fluid of H9N2 (A/chicken/Iran/772/1998)-infected chicken embryo. The neutralization index (NI) is used to evaluate the efficacy of the applied product in virus inactivation. The extract shows a maximum non-toxic concentration of > 7.7, which has a considerable effect, while amantadine shows 2.2 and has no antiviral effect. The dose-dependent inhibitory effect of the extracts identified using quantitative real-time/polymerase chain reaction (qRT/PCR) and hemagglutination (HA) testing. The EF and SAM, in the ratio of 2:1 combined to form 0.6 ml, produce virucidal effect against 0.6 ml H9N2, equivalent to 500 EID_50_/0.1 ml [49].

The author and his research team verified the effect of the extracts and amantadine on H9N2-infected Cobb broiler chicks (A/chicken/Iran/772/1998) and divided them into groups 1,2,3,4, and 6. Echinaforce® (EF), amantadine, and Sambucol® (SAM) were administered for groups 1, 2, and 3 for seven days from the 8-h-PI. Group 5 acted as a negative control. Group 4 was positive control without administering drugs. Group 6, as a prophylactic group, and EF gave for five days in pre-infection with 8-h, no treatment, and five days in post-infection. The HI antibody titer analysis showed significant antibody production in all groups except the uninfected negative control group. Virus titration studies have shown that the amount of virus excreted in the feces is higher than in the trachea. The virus titer in the feces of EF groups 1 and 6 showed nil loads on 3 d.p.i. compared to other groups. Groups 2, 3, and 4 showed low titer on 9 d.p.i. and nil loads on 12 d.p.i. The tracheal assay showed SAM and amantadine-treated groups without virus load at 3, 6, and 12 d.p.i.; the virus load constantly exists in EF and positive control groups in all days of post-infection [50].

Rauš et al. conducted a randomized, double-blind, double-dummy, multicenter, controlled clinical trial in 473 patients with flu symptoms ≤ 48 h, of which 237 patients used EF, 236 patients received oseltamivir. One, five, and ten days later, the results showed that the symptom reduction was 1.5% and 4.1%; 50.2% and 48.8%; and 90.1% and 84.8%, respectively, indicating that the effect of EF is not inferior to oseltamivir. EF hot drink reduces the complications (*P* = 0.076) with fewer adverse effects like nausea and vomiting [51].

The old stock of EF also has the retentive power against influenza viruses. These were demonstrated by Schoop et al. by checking the EF with various modes as newly prepared, concentrated spissum, and an eight-year-old tinctures against H7N9 (A/Anhui/1/2013) and observed the IC_50_ as 7.01 µg/ml, 27.31 µg/ml, and 9.37 µg/ml [52].

Generally, viral infections create permissive circumstances for bacterial co-infections. In specific, Influenza A (H3N2) induces inflammation and secondary bacterial infections to cause pneumonia. Vimalanathan et al. observed the inhibitory effect of EF in H3N2 (A/Victoria/H3N2) induced adhesion of bacteria NTHi (nontypeable *Haemophilus influenza*), and *Staphylococcus aureus* in BEAS-2B cells and found EF dilution of 1:200 and 1:1600 are non-cytotoxic and retain the cell viability. Influenza virus up-regulates the bacterial (NTHi) receptor intracellular adhesion molecule-1 (ICAM-1), and EF significantly reduces the ICAM-1. The extract at a concentration of 80 μg/ml reduced the expression of the ligating receptor PAFr and fibronectin, thereby preventing the virus-induced invasion of NTHi and *S.aureus*. The virus-stimulated cytokine productions (IL-6, IL-8) were remarkably down-regulated by EF in a dose-dependent manner, and the contribution of modulators such as toll-like receptor (TLR-4) and NFκB p65 suppressed by EF at 80 μg/ml concentrations [53].

## EPs® 7630 (Umckaloabo®)

### Brief view

EPs® 7630 (Umckaloabo®) fortified with polyphenolic compounds, especially prodelphinidins as the major constituents [54] acquired from the roots of *Pelargonium sidoides* DC. Pharmacognosy of the herbal product is by Dr. Willmar Schwabe Pharmaceuticals, Karlsruhe, Germany [55, 56].

### Mode of action

Michaelis et al. checked the efficiency of EPs® 7630 against various types of viruses like respiratory syncytial virus (RSV), adenovirus, parainfluenza virus, human rhinovirus, influenza virus, coronavirus, and coxsackie virus. The influenza virus strains like A/New Caledonia/20/99 (H1N1), A/California/7/2004 (H3N2), and A/Thailand/1(Kan-1)/04(H5N1) and cell lines like MDCK, Vero were chosen for the influenza virus culture. The authors not observed the decrease in cell viability but the cytopathogenic (CPE) effect in H1N1, H3N2 not in H5N1 by the extract EPs® 7630 of up to 100 μg/ml. Moreover, the authors point out, the cytoprotective effect is the result of the prevention of CPE, which in turn reflects the prevention of virus replication, and thus the product is suggested for the treatment of acute bronchitis [55].

Theisen et al. examined pronounced inhibitory activity of EPs® 7630 against A/Puerto Rico/8/34-NS116-GFP, pandemic H1N1 A/Luxembourg/46/2009, H1N1 A/Puerto Rico/8/34, seasonal H3N2 A/Luxembourg/01/2005, seasonal oseltamivir-sensitive H1N1 A/Luxembourg/663/2008, seasonal oseltamivir-resistant H1N1 A/Luxembourg/572/2008 along with adenovirus, measles virus. In post-incubation, the extract CC_50_ value (557 μg/ml) lowers than the inhibitory EC_50_ value (above 50 μg/ml) and inhibits the early stage of viral entry along with hemagglutination and neuraminidase inhibitory activity. These antiviral effects were not observed in the adeno and measles virus. Meanwhile, the extract does not generate resistant viruses. Further constituents analysis of the extract reveals polyphenols, especially prodelphinidins, with its oligo and polymers gallocatechin and its stereoisomers epigallocatechin, responsible for the anti-influenza activity. The in vivo study was conducted by infecting the mice with the influenza strain A/Puerto Rico/8/34 and administering the extract by inhalation. After the treatment, the authors observed improved survival time along with increasing body weight without toxic effects [56].

## Ladania067®

### Brief view

Ladania067**®** is another therapeutic option available for the treatment and prevention of influenza infections. Ladania067**®** is the water-soluble extract made from the leaves of *Ribes nigrum* L. Currently, Dr. Pandalis NatUrprodukte GmbH & Co. KG, Glandorf, Germany is administrator of the extract [57, 58].

### Mode of action

Ladania067**®,** fortified with anti-influenza activity, was demonstrated by Haasbach et al. through their in vitro and in vivo studies. The extract did not exhibit a cytotoxic effect on MDCK, A549, and HeLa cells in the range of CC_50_ 0–1 mg/ml, whereas for human PBMCs (human peripheral blood mononuclear cells) CC_50_ ranges from 0.5 ± 0.3 mg/ml. As the cytotoxic concentration was found to be high in PBMCs cells, the author scrutinizes the activity of the extract in lymphocyte proliferation and observed the null effect. The in vitro anti-influenza activity of the extract against the strain A/Regensburg/D6/09 (H1N1) showed an EC_50_ value as 49.3 ± 1.1 ng/ml and the effect notified at pre- and direct incubation stage, not in post-infection, indicates an early stage of viral inhibition. Dose-dependent intranasal application, 500 μg of the extract showed an 85% reduction in the lung virus titers, thereby reducing progeny virus. Survival experiments exhibit a stabilization of body weight by day 6 p.i., and decrease symptoms of the disease by day 7 p.i., thus safeguards from death [57].

The anti-influenza potential of Ladania067® was tested through in vitro and in vivo studies by Ehrhardt et al. The influenza strains like A/FPV/Bratislava/79 (FPV) (H7N7), A/Puerto Rico/8/34 (PR8, H1N1rec), and A/Nordrhein-Wesfalen/173/2009 (H1N1pan) harboring the oseltamivir resistance mutation NA (H275Y) [20] were selected and propagated in MDCK cells. Ladania067® dose-dependent treatment show reduction of progeny viruses in these infected cell line. The extract, even at the effective dose of 100 μg/ml, did not display cellular hindrance like affecting cell morphology, viability, metabolism, or proliferation, without inducing virus resistance. The internalization of the influenza virus by the cell regulated by epidermal growth factor receptor (EGFR) has been blocked by Ladania067®. The influenza strain A/Puerto Rico/8/34 (H1N1rec)-infected mice intranasally administered with the extract using COAALA Mouse Aerosol Application System showed reduced viral lung titer without bodyweight reduction [58].

## Mentofin®

### Brief view

EWABO, Wietmarschen, Germany is the commercial manufacturer of Mentofin®. It is a natural water-soluble concentrate combined with essential oils extracted from Eucalyptus and Peppermint, in the combination of 10% menthol, 10% eucalyptus oil, 33% liquid builders, and 47% saponins [59].

### Mode of action

Avian influenza viruses are zoonotic pathogen that infect humans and other mammals without specific specification [60]. This is achieved by their genetic reassortment, producing new variant strains that generate a pandemic scene [61]. In poultry, it creates the worst economic impact with 100% mortality [62]. Hence, actions are needed for the efficient control and spread of the disease. A commercial product like Mentofin® has been successfully launched against avian influenza viruses, and their details are given below.

Barbour et al. conducted an in vivo study on seven groups of seventy-one-day-old broiler chicks against controlled challenges by the bacterium mycoplasma gallisepticum (MG) and/or avian influenza virus H9N2. For the one week of age birds after post-treatment of MG and/or H9N2, mentofin® of 1 ml was administered intra-esophageally for 6 days. Following this histopathological study conducted in the tracheal tissue lesions revealed a significant reduction (*P* < 0.05) in tracheal deciliation with degeneration of goblet cells, mucosal hypertrophy along with mucus accumulation, and heterophil infiltration [63].

Barbour et al. analyzed the safety and antiviral efficacy of five different concentrations of Mentofin® (2.78 × 10–3, 2.78 × 10–2, 2.78 × 10–1, 2.78, 13.9, and 27.8%) with or without 1% skim milk against H9N2 and Newcastle disease virus (NDV) in 10-day-old embryonated eggs. The result revealed that all five concentrations are safe and did not affect the survival of the embryos. Mentofin® at the concentration of 2.78 × 10–1% and 13.9% showed virucidal effect against H9N2 and NDV at the contact time of 30 min without skimmed milk. The authors argued that skimmed milk organic matter act s as a neutralizing agent for Mentofin® [64].

The same author, along with other investigators, studied the effect of Mentofin® against respiratory pathogen including H9N2 and other infectious agents like infectious bursal disease virus (IBDV), Newcastle disease virus (NDV), mycoplasma gallisepticum (MG), and infectious bronchitis virus (IBV). For this study, eighty-day-old broiler chicks were vaccinated with IBDV and NDV and classified for four treatments. Treatments 1 and 2, at 7 days of age, were challenged with MG at 28 days of age, challenged with H9N2 and IBV. Treatment 3 was grouped as unchallenged, whereas treatment 4 was used as control. Mentofin® was administered to the treatment 2 and 3 groups and observed the mortality reduction along with an improved liver function. The chickens in the second group were challenged with lesions and decreased creatinine. The unchallenged treatment group 3 showed an improved feed conversion ratio (FCR). It is reported that H9N2 and IBDV have reduced immune reactions, whereas NDV obtains the vaccine boosting immunity [65].

Sultan et al. selected and assigned two hundred and forty SPF day-old broiler chicks for five treatments, as A, B, C, D, and E. A was the negative control. B, C, D, and E groups were challenged with H9N2. D and E were vaccinated with the killed H9N2 vaccine. The birds in the treatments of C and D were intermittently administered with Mentofin® in drinking water. The results showed that vaccinated, challenged, and Mentofin®-treated D birds enhanced their immune potential by exhibiting low heterophil, low enzymatic blood characteristics, and low FCR, as well as gaining weight [66].

## Oximacro®

### Brief view

Oximacro®, extract of berries of *Vaccinium macrocarpon* Aiton, a trademark of Biosfered S.r.l., Turin, Italy. The extract is rich in proanthocyanidins PAC-A (86.72% ± 1.65) and other phytochemicals such as delphinium, anthocyanin, quercetin, and isorhamnetin [67].

### Mode of action

The author, Luganini, and his colleagues first explored the chemical composition analysis of Oximacro®, observing that the accumulation of proanthocyanidin PAC-A dimer is higher than that of the trimer, and they play a crucial role in anti-influenza inhibitory activity. Oximacro® used in MDCK cells to inhibit the replication of IAV (A/Puerto Rico/8/34) and IBV (B/Lee/40) with IC_50_ of 4.5 ± 0.2 µg/ml and 4.5 ± 0.5 µg/ml and CC_50_ value as 141 ± 0.8 µg/ml and their observed selectivity index (CC_50_/ IC_50_) were 31.1 and 31.3 without prevailing cytotoxic effect. The extract binds to the hemagglutinin (HA) of the viruses, thereby preventing its attachment and entry into the cells. The direct interaction between HA and the dimeric PAC-A (PAC-A2) of the extract was evident through fluorescence spectroscopy and computational docking studies [67].

## Sambucol® and its related products

### Brief view

Sambucol® (SAM) is a standardized black elderberry extract (Sambucus nigra L.). Razei Bar Company in Jerusalem, Israel and PharmaCare Europe Ltd. in West Sussex, England are the manufacturer of Sambucol®. The unique formulation of sambucol by Razei Bar has 38% of the extract, with trace amounts of raspberry extract, glucose, citric acid, and honey. PharmaCare syrup products contain 1.9 g of extract per 5 ml [49, 68–70]).

The 175 mg elderberry extract prepared in the form of a lozenge is a proprietary extract of HerbalScience Singapore Pte. Ltd. The elderberry liquid extract named Rubini is a proprietary product produced by BerryPharma AG, Leichlingen, Germany. The concentrated elderberry juice (CJ-E) is manufactured by Bayernwald, Germany.

### Mode of action

Zakay-Rones et al. conducted in vitro studies using Sambucol® (SAM without additives) against the human influenza A viruses H1N1 (A/Texas 36/91, A/Singapore 6/86), H3N2 (A/Shangdong 9/93, A/Beijing 32/92), and B viruses (B/Panama 45/90, B/Yamagata16/88, B/Ann Arbor 1/86) along with H1N1 of animal strains (A/Sw/Ger 2/81, A/Tur/Ger 3/91, and A/Sw/Ger 8533/91) cultured in MDCK cells. The extract at the dilution of 1:4 inhibited hemagglutinin for A/Beijing 32/92 (H3N2), A/Singapore 6/86 (H1N1), B/Panama 45/90, and B/Yamagata 16/88 in 1 h of short incubation. High dilutions (1:8 to 1:16) with a high incubation period of up to 16 h potently inhibit HA of all viruses. The addition of SRBC (sheep red blood cells) with the extract confirmed that the binding mode is the HA of the virus. The extract inhibits the replication of the viral strains in a dose-dependent manner, thereby reducing the cytopathic effect (CPE). The dilution of 1:8 produces complete CPE when compared with 1:16. Along with the in vitro experiments, the authors conducted a double-blind placebo-controlled study during an outbreak of influenza B/Panama in 1993 by administering the extract for 6 days among forty non-vaccinated individuals who classified into three categories based on the symptoms like fever > 38 °C, myalgia, nasal discharge, and cough, which displayed in 24 h duration. The sambucol treatment group showed a nearly 90% complete recovery within 2 to 3 days, while the placebo showed *P* < 0.001 within at least 6 days [68].

Zakay-Rones, with his team of researchers, conducted the randomized, double-blind, placebo-controlled study by administering 60 ml of sambucol for five days to sixty patients not belong to the high-risk group, but affected with fever ≥ 38.0 °C and at least one respiratory influenza symptom. Patients treated with sambucol showed significant improvement within 3–4 days, while placebo only reached it after 7–8 days [69].

Sambucol ® was applied to MDCK cells infected with avian influenza virus H5N1 (NIBRG-14) to determine its anti-influenza efficacy and observed that it was in a dilution of 1:8 and above does not produce cytotoxic effects. The therapeutic index calculation shows that the direct inhibitory activity of Sambucol ® at 1:4 and 1:8 dilutions can reduce 99% of the virus by at least 2.0 log10 TCID/ml within 30 s [70].

The combinatory effect of Sambucol® along with Echinaforce® and amantadine produces virucidal effect against H9N2 [49, 50].

Burge et al. analyzed the prophylactic effect of Sambucol® in a chimpanzee colony by oral supplementation of 10 ml daily during the onset of flu season and 15 ml supplement the dose for animals that have symptoms of infection. Studies have shown that animals that consume the extract have reduced duration and symptomatic effects by 70% [71].

A pilot randomized, double-blind, and placebo-controlled clinical study of 64 patients between the ages of 16 and 60 who had flu symptoms for less than 24 h, divided into two groups. For the first group, four slowly dissolving elderberry extract lozenges and, for the second, placebo administered for two days. The lozenges treatment revealed the reduction of symptoms with significant improvement within 24 h of treatment, and complete eradication of infectious symptoms within 48 h recorded, whereas placebo treatment remains unchanged or with the worst condition. The lozenge group showed almost 60% symptom relief and 28% complete recovery without side effects [72].

Rubini® is a standardized elderberry liquid extract, diluted 1:100, and its potential inhibitory effect on H5N1 is 30% (A/Thailand/KAN-1/2004) and 25% on B (B/Massachusetts/71), which is cultured in MDCK cells without cytotoxicity [73].

Elderberry juice concentrate (CJ-E®) has anti-influenza effects in both in vitro and in vivo analyses. Although in vitro studies did not give satisfactory results, they were considered weak, but in vivo produced gratifying results. For in vivo experimental studies, BALB/c mice were infected with H1N1 (A/NWS/33), and the compound was administered three days before infection and seven days after infection. Administration of 5 mg/d and 1 mg/d extracts showed a weight loss of 15.4% and 19.7%, as well as a reduction in virus, and the antibody titer in the BALF (bronchial alveolar lavage fluid) of infected mice increased 1.8–1.6 times. Indeed, the extract stimulates the immune response by increasing IgA and IgG antibodies in the BALFs and sera. Further chemical analysis of CJ-E revealed that the high molecular weight fractionated compounds Fr. I, Fr. II, including acidic polysaccharides, may be responsible for the anti-influenza activity [74].

### Safety profile

As far as influenza is concerned, the above said commercial products recorded with minimum or free of adverse side effects. COLD-FX® (CVT-E002) verified with high safety profile [75], however, fewer effects like the gastrointestinal, nervous, cardiovascular problem with the limitation for pregnant or breast-feeding women, and showed the interaction effect of the drug warfarin [76]. CYSTUS052® proved effective without toxic side effects [45]. Echinaforce® has established a similar effect as oseltamivir and reduces adverse side effects such as induction of allergic reactions, leukopenia, and autoimmune diseases [7, 47, 77]. EPs® 7630 (Umckaloabo®) recorded < 15% of 79,143 subjects with reduced side effects such as gastrointestinal and skin rashes and showed no contradictory effect on pregnant or lactating women [78]. Ladania067® recommended for prophylaxis without showing awful side effects or allergic reactions [58]. Mentofin® and Oximacro® have proved to be effective against anti-influenza activities, and their adverse effects have not been documented so far [59, 67]. Sambucol® was used as prophylaxis, without exposing any side effects on healthy adults [49, 68, 69, 79], but the impact on high-risk patients needs to be studied [69]. In general, these commercial products are the alternate, important options for influenza prevention, with mild or non-toxic side effects.

## Conclusion

To handle the dreadful condition of influenza remains a challenge. Traditional medicine offers an alternate remedy for the situation. Plants enriched with antiviral sources proved to be anti-influenza agents. Based on the evidence-based scientific support, many commercial products successfully launched and demonstrated their efficacy against influenza. This intriguing aspect provides a source to find new effective and viable products soon.

## Data Availability

All information provided in the manuscript is obtained from the references.

## Abbreviations

ARI: Acute respiratory infections
BALF: Bronchial alveolar lavage fluid
CC_50_: Half maximal cytotoxic concentration
CJ-E: Elderberry juice concentrates
CPE: Cytopathic effect
EC_50_: Half maximal effective concentration
EF: Echinaforce®
EGFR: Epidermal growth factor receptor
FCR: Feed conversion ratio
HA: Hemagglutinin
HeLa: Human epithelioid cervical carcinoma
IBDV: Infectious bursal disease virus
IBV: Infectious bronchitis virus
ICAM-1: Intracellular adhesion molecule-1
IFN-γ: Interferon gamma
IgA: Immunoglobulin A
IgG: Immunoglobulin G
IL-2: Interleukin
LCCUs: Laboratory-confirmed clinical URIs
LCII: Laboratory-confirmed influenza illness
MDCK: Madin-Darby bovine canine kidney cells
MG: Mycoplasma gallisepticum
NDV: Newcastle disease virus
NFκB: Nuclear factor kappa B
NI: Neutralization index
NK: Natural Killer
NTHi: Nontypeable *Haemophilus influenza*
p.i.: Post-infection
PAC: Proanthocyanidins
PAFr: Platelet-activating factor receptor
PBLs: Peripheral blood leukocytes
PBMCs: Peripheral blood mononuclear cells
Proanthocyanidins A: PAC-A
qRT/PCR: Quantitative real-time/polymerase chain reaction
RSV: Respiratory syncytial virus
SAM: Sambucol®
SPF: Specific pathogen free
SRBC: Sheep red blood cells
TLR-4: Toll-like receptor-4
TNF-α: Tumor necrosis factor α
URIs: Upper respiratory infections
WHO: World Health Organization
